# Ho-SiAlON Ceramics as Green Phosphors under Ultra-Violet Excitations

**DOI:** 10.3390/ma15196715

**Published:** 2022-09-27

**Authors:** Yuwaraj K. Kshetri, Bina Chaudhary, Dhani Ram Dhakal, Soo Wohn Lee, Tae-Ho Kim

**Affiliations:** 1 Research Center for Green Advanced Materials, Sun Moon University, Chungnam 31460, Korea; 2 Department of Fusion Science and Technology, Sun Moon University, Chungnam 31460, Korea; 3 Department of Environment and Bio-Chemical Engineering, Sun Moon University, Chungnam 31460, Korea

**Keywords:** SiAlON ceramics, downshifting luminescence, phosphors, concentration quenching

## Abstract

In most inorganic phosphors, increasing the concentration of activators inevitably causes the concentration quenching effect, resulting in reduced emission intensity at a high level of activator doping and the conventional practice is to limit the activator concentration to avoid the quenching. In contrast, SiAlON ceramics preserve their chemical composition over a very wide range of doping of activator ions, which favors the adjustment and optimization of the luminescence properties avoiding concentration quenching. Here, we investigate the photoluminescence properties of Ho-doped SiAlON (Ho-SiAlON) ceramics phosphors prepared by the hot-press method. Ho-SiAlON ceramics show strong green visible (554 nm) as well as infrared (2046 nm) broadband downshifting emissions under 348 nm excitation. It is shown that there is no concentration quenching, even at a very high level of Ho doping. The emission intensity of the 554 nm band increased two-fold when the Ho concentration is doubled. The results show that the Ho-SiAlON ceramics can be useful for efficient green phosphors.

## 1. Introduction

SiAlON ceramics are known to have an incomparable balance of mechanical and thermal properties [1,2] that enable them to resist extreme thermal shock and keep mechanical integrity in temperatures as high as 1673 K (1400 °C). For this reason, SiAlON ceramics are preferred for use in refractory applications over traditional ceramics, such as zirconia (ZrO_2_) and alumina (Al_2_O_3_). SiAlON ceramics are generally prepared at high temperatures using the solid-state reaction technique. The precursor is an appropriate mixture of α-Si_3_N_4_, AlN, Al_2_O_3_, and a small amount of oxides of Ca, Mg, or lanthanides (Ln^3+^) as a sintering additive [3]. The Ln^3+^ ion that facilitates the sintering of α-Si_3_N_4_ [4] helps to keep the charge neutrality in the α-SiAlON crystal during the preferential substitutions of (Si, Al) and (N, O) in the lattice [5]. Moreover, Ln^3+^ ions also impart the α-SiAlON ceramics with efficient photoluminescence [6,7]. α-SiAlON ceramics have been reported as efficient inorganic remote phosphors for white light emitting diodes (WLEDs) [8,9].

Generally, an increase in the activator concentration should be accompanied by an increase in the intensity of the emitted light due to increased absorption efficiency of the excitation energy. However, in most inorganic phosphors, increasing the concentration of activators inevitably decreases the emission intensity [10,11,12,13,14,15], a phenomenon called concentration quenching. Concentration quenching of the emission is attributed to the increased probability of energy migration between the luminescent centers as the distance between the luminescent centers becomes shorter with increasing activator concentration [16]. Conventional practice is to limit the activator concentration to avoid concentration quenching. In this regard, SiAlON ceramics preserve their chemical composition over a very wide range of doping of activator ions, which favors the adjustment and optimization of the luminescence properties without suffering from concentration quenching.

In addition to the high level of doping ability of activators, the thermal stability of the host is also very important for stable optical performance. This is because luminescence stability is closely associated with the structural stability of the phosphor. The stronger the structural rigidity, the better the thermal stability of the host, and hence, stable the luminescence. Many of the efficient glass-based conventional phosphors [17] have low thermal stability. In such phosphors, the lattice thermal expansion due to heat may change the local environment of the crystal site occupied by the Ln^3+^ ion [18,19], ultimately causing thermal quenching of the emission bands. In this regard, phosphors with a very low thermal expansion coefficient are preferred for stable luminescence. SiAlON ceramics are known to have a very low thermal expansion coefficient [1], and therefore, have high thermal stability, even at extreme temperatures. Therefore, studies on the Ln^3+^-doped SiAlON phosphors have a considerable research value. The holmium ion (Ho^3+^) can be efficiently excited in the ultraviolet (UV) and blue spectral range giving strong green luminescence due to the 4*f*-4*f* transition of the Ho^3+^ ion. Hence, Ho-SiAlON ceramics can be potential green phosphors.

In this report, we synthesize Ho-SiAlON ceramics by the hot-press technique and investigate their photoluminescence properties under UV excitations. The ceramic samples are characterized for phase and morphology using XRD and SEM, respectively. The valence state of the dopant has been confirmed by the XPS analysis. We study Ho concentration-dependent photoluminescence of Ho-SiAlON ceramics and find that these ceramics have a high level of doping tolerance of Ho ions and do not suffer from concentration quenching. It will be shown that the ceramics can be useful for green phosphors.

## 2. Experimental Section

A series of Ho-SiAlON ceramics were obtained by the hot-press sintering method. Sample compositions were designed according to Ho_x_Si_12−m−n_ Al_m + n_O_n_N_16−n_; x = m/v, where x is solubility and v is the valency of the metal cation Ho [3]. The starting m and n parameters and precursors composition are given in Table 1. Four different sets of m and n parameters were chosen as listed in Table 1. α-Si_3_N_4_ (UBE Co., Tokyo, Japan), AlN (Tokuyama Co., Tokyo, Japan), Al_2_O_3_, and Ho_2_O_3_ (High purity chemicals Co LTD., Saitama, Japan) were used as starting materials. The samples were hot-press sintered at 1850 °C, as described elsewhere [20,21]. The samples were re-sized and polished for various characterizations.

The X-ray diffraction (XRD) spectra were obtained using Cu K_α_ radiation (Rigaku D/Max 2200HR diffractometer, Akishima, Tokyo, Japan) at a scanning rate of 1° per minute in the 2θ range of 10° to 90°. The X-ray photoelectron spectra (XPS) were recorded by Versa Probe spectrometer (PHI 5000, Ulvac-PHI, Kanagawa, Japan) with monochromated Al K_α_ (1486.6 eV) as an X-ray source. The transmission electron microscopy (TEM) images and energy-dispersive spectra (EDS) were recorded by using TEM (JEM-4010, JEOL, Tokyo, Japan). Absorption spectra in the 200–2500 nm range were measured by a V-570 spectrophotometer (Jasco International Co. LTD., Tokyo, Japan). The photoluminescence spectra were obtained using an FLS980 spectrometer (Edinburgh Instruments, Edinburgh, UK). A 400 Watt Xenon lamp was used for UV excitation in CW mode and a microsecond flash lamp (µf2, Edinburgh Instruments, Edinburgh, UK) was used for pulsed excitation.

## 3. Results and Discussion

### 3.1. Phase and Microstructure Analysis

**Figure 1:**
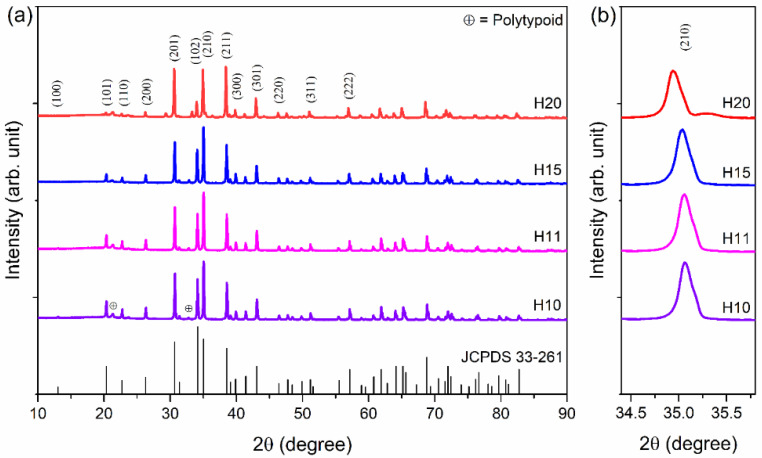
(**a**) XRD spectra of different Ho-SiAlON ceramic samples of composition presented in Table 1. The line spectrum on the bottom axis is the XRD spectra of the Ca-α-SiAlON of JCPDS 33-261. (**b**) Enlarged view of the (210) XRD peak.

Figure 1 displays the XRD spectra of all the sintered samples and shows that the Ho-SiAlON samples have similar XRD patterns. The peaks have been indexed with reference to the XRD spectra of the Ca-α-SiAlON (JCPDS 33-261). The chosen m and n parameters are within the solubility limits of α-SiAlON solid solutions [22,23]. However, there are a few minor peaks of the SiAlON polytypoid phase [24]. The samples show that there is a gradual shift of the (210) peak (Figure 1b) towards a smaller angle with the m parameter increasing from m = 1.0 to m = 2.0. With an increasing m value, the content of Ho increases (Table 1) in the sample. Hence, the shifting of the (210) plane with increasing m value can be due to the increase in the unit cell size [23].

**Figure 2:**
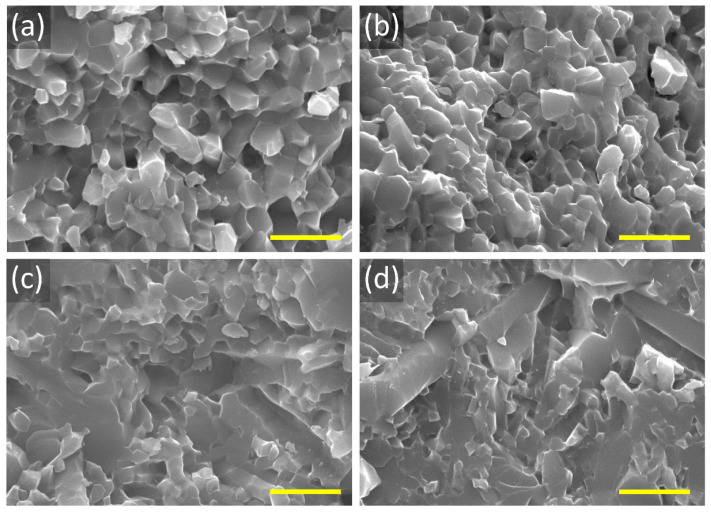
SEM images of the fracture surface of the Ho-SiAlON ceramics (**a**) H10, (**b**) H11, (**c**) H15, and (**d**) H20. The scale bar is 5 µm in all the images.

**Figure 3:**
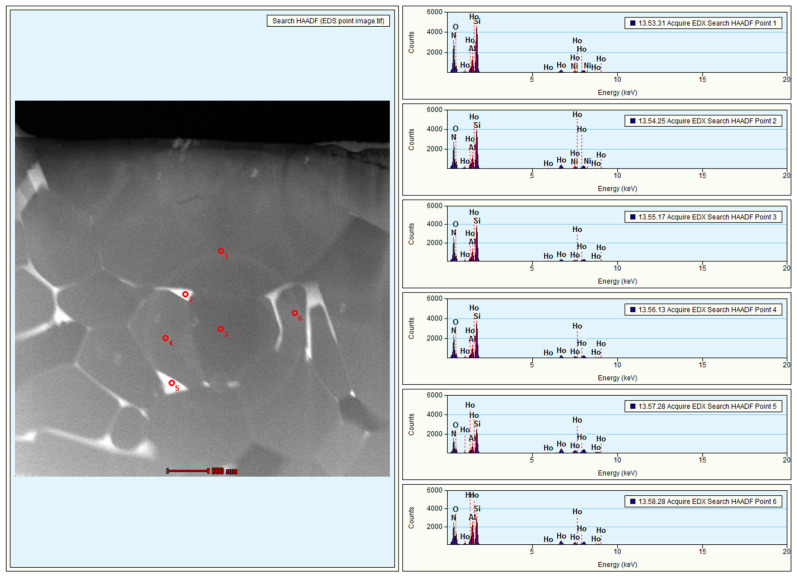
TEM micrograph of H15 sample (**left**) and quantitative EDS spectra (**right**) of the points indicated in the TEM micrograph on the left. Points 1, 3, 4, and 6 correspond to inside the grains, and points 2 and 5 correspond to the triple junctions, respectively.

**Figure 4:**
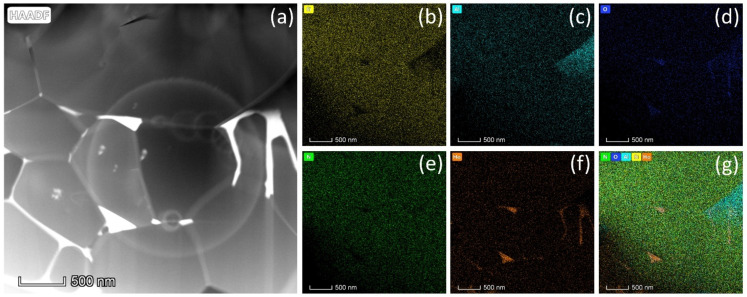
Elemental mapping diagram of H15 sample. (**a**) HAADF image. Images (**b**–**f**) correspond to the mapping spectra of Si, Al, O, N and Ho, respectively, of the region indicated in (**a**). (**g**) Superposition of the elemental spectra of (**b**–**f**).

Figure 2 shows the SEM images of the fracture surface of all the samples. A fully dense α-SiAlON microstructure without pores is evident from the micrograph in Figure 2. It can be noticed that the grain morphology changes with increasing Ho concentration. Samples H10 and H11 are composed of isometric polyhedral grains, while more elongated grains can be seen in samples H15 and H20. The fracture mode also appears to be different in different samples. The transverse grain fracture and grain pull-out can be seen in samples H10 and H11, while lateral grain sliding can be observed in samples H15 and H20. Additionally, the TEM micrograph of sample H15 in Figure 3 shows the grains of the α-SiAlON phase with grain sizes ranging from sub-micron to a few micrometers. Moreover, the EDS spectra of different points across the sample show the presence of all the elements. Figure 4 shows the high-angle annular dark-field (HAADF) TEM image and corresponding elemental mappings of H15 sample. Homogeneous distribution of Si, Al, O, and N across the matrix of Ho-α-SiAlON can be seen. However, a relatively higher concentration of Ho ions at the triple junction (point 2 and point 5) can be observed in Figure 4f,g. The higher concentration of Ho ions at the triple junction is due to the segregation of the Ho ions, which are not incorporated into the α-SiAlON lattice during sintering.

### 3.2. XPS Analysis

**Figure 5:**
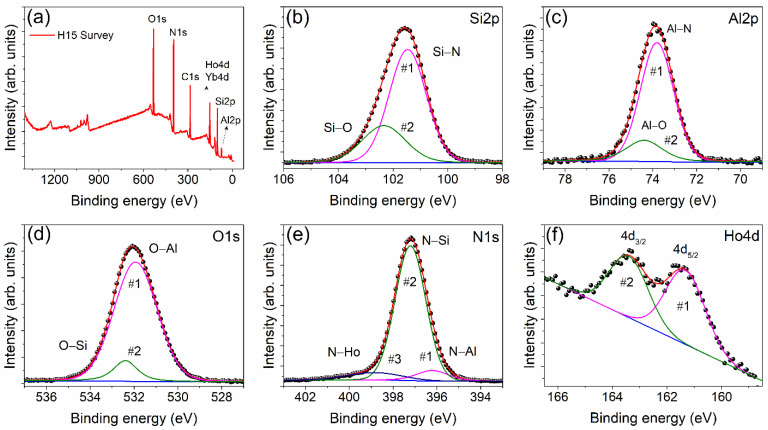
(**a**) XPS survey spectra of H15 sample. (**b**–**f**) are the high-resolution XPS spectra of constituent elements in the H15 sample. Dots represent the observed data points and continuous lines represent the fitted graph.

XPS has been extensively used for the characterization of Si_3_N_4_-based thin films [25,26], powders [27], and sintered materials [28]. However, XPS studies on the hot-pressed α-SiAlON ceramics are limited [29]. The XPS survey spectra and high-resolution elemental spectra of the H15 sample are shown in Figure 5. The peaks corresponding to each constituent element have been fitted and the results are presented in Table 2. A carbon peak at a binding energy of 284.83 eV observed in the survey spectra (Figure 5a) was used for the calibration.

In Figure 5b, the Si 2p peak has two sub-peaks. Since the binding energy of the photoelectrons increases with the increase in electronegativity of the nearest neighbors of an atom, the Si 2p line can be expected to be at high binding energy due to the high electronegativity of oxygen atoms with respect to that of nitrogen atoms [30]. Therefore, the dominant sub-peak of lower binding energy corresponds to the Si-N bond in the Si_3_N_4_ matrix [31], while the less intense sub-peak of higher binding energy corresponds to the Si-O bond in SiO_2_ [32] that exist in the grain boundary glassy phase of the SiAlON ceramics [33]. In Figure 5c, the Al 2p scan has two sub-peaks. The peak with lower binding energy corresponds to the Al-N bond that originates from AlN-rich phases in SiAlON ceramics and the peak with higher binding energy can be assigned to the Al-O bond [34]. The Al-O bond appears as a result of the partial Al(Si)-O(N) substitution in the Si_3_N_4_ matrix during the formation of the α-SiAlON lattice [3]. In Figure 5d, the O1s scan consists of two peaks. The intense peak at lower binding energy can be assigned to the O-Al bond that results due to Al(Si)-O(N) substitution as discussed earlier. The less intense peak at higher binding energy is due to the O-Si bond of the intergranular glassy phases. The N1s peak consists of three sub-peaks as shown in Figure 5e. The weak sub-peak at the lowest binding energy corresponds to the N-Al, while the intense central peak corresponds to the N-Si bonds in the α-SiAlON lattice because the N1s binding energy for AlN is less than that for Si_3_N_4_ [35]. In the α-SiAlON crystal, the Ln^3+^ dopant (Ho^3+^) occupies the seven-fold coordination site [5,21] for the charge compensation which arises due to the partial Al (Si)-O (N) substitution in the formation of the SiAlON lattice [3]. Hence, it is suggested that the broad sub-peak with the highest binding energy in Figure 5e corresponds to the N-Ho bond. The XPS spectrum of Ho4d in Figure 5f is the characteristic of the trivalent Ho ion [36]. Hence, it can be inferred that the Ho ion exists in a 3+ valance state in the α-SiAlON lattice.

### 3.3. Absorption Spectra:

**Figure 6:**
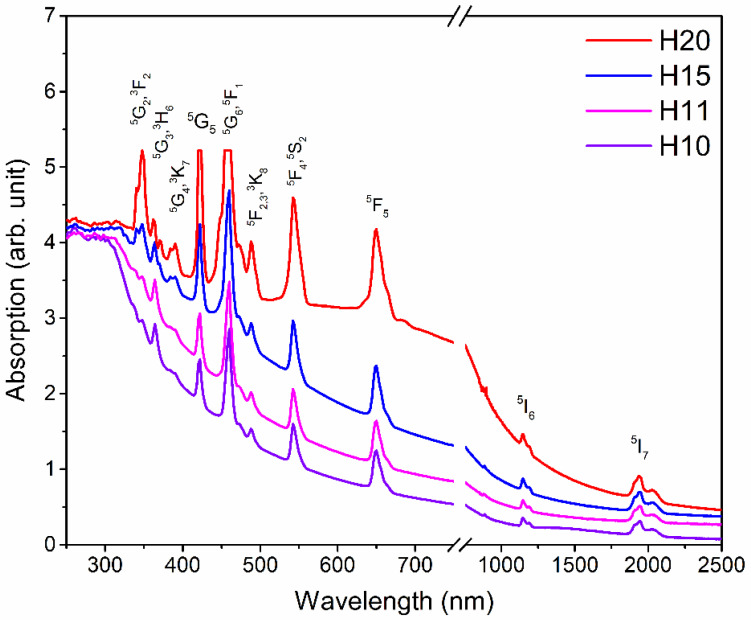
Absorption spectra of Ho-SiAlON samples.

The absorption spectra of all the Ho-SiAlON samples are shown in Figure 6 in the range of 250–2500 nm. All the samples show the characteristic absorption peaks of the Ho^3+^ ion. The absorption peaks at 340, 348, 364, 390, 422, 460, 488, 543, 650, 1148, and 1945 nm are assigned, respectively, to the transitions to the excited states ^3^F_2_, ^5^G_3_, ^3^H_6_, ^5^G_4_ + ^3^K_7_, ^5^G_5_, ^5^F_1_ + ^5^G_6_, ^5^F_2_,_3_, ^5^F_4_ + ^5^S_2_, ^5^F_5_, ^5^I_6_, and ^5^I_7_ from the ground state ^5^I_8_ of Ho^3+^ ion [37]. Below 320 nm, an ultraviolet absorption edge exists in all the samples, which can be assigned to the charge transfer within the Si(Al)-N(O) network in the Ho-SiAlON matrix [38].

### 3.4. Downshifting Photoluminescence in Ho-SiAlON Ceramics

**Figure 7:**
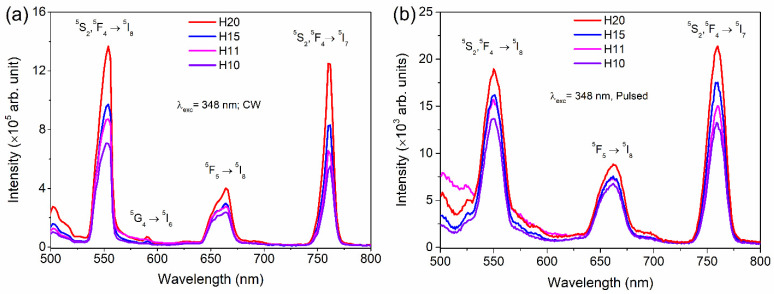
Downshifting spectra of Ho-SiAlON samples under 348 nm (**a**) CW excitation and (**b**) pulsed excitation.

Emission spectra of Ho-SiAlON samples in the visible spectral range under 348 nm continuous wave (CW) as well as pulsed excitation are shown in Figure 5a,b, respectively. The 348 nm excitation corresponds to the ^5^I_8_ → ^5^G_3_ absorption transition of Ho^3+^ ions (Figure 10). There are three sharp and strong emission bands at 554 nm (green), 664 nm (red), and 760 nm (near-infrared), which are assigned to the transitions ^5^F_4_, ^5^S_2_ → ^5^I_8_, ^5^F_5_ → ^5^I_8_, and ^5^F_4_, ^5^S_2_ → ^5^I_7_, respectively of the Ho^3+^ ion [39]. A weak band at 590 nm is assigned to the ^5^G_4_ → ^5^I_6_ transition. Intensities of the 554 and 760 nm emission bands increase with the increase in Ho concentration. As shown in Figure 7a, a nearly two-fold increase in the intensity of the 554 nm emission band is observed for the H20 sample (Ho-concentration 18.068 wt.%) as compared to the H10 sample (Ho-concentration 9.791 wt.%). The existence of these emission bands is an indication that the Ho^3+^ ions have been incorporated into the SiAlON lattice.

**Figure 8:**
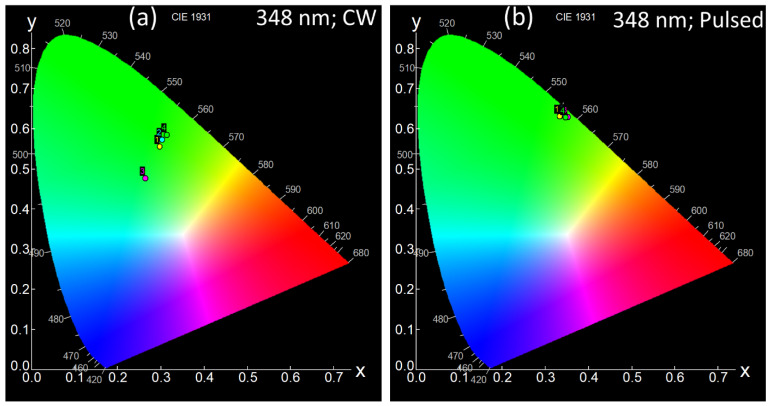
CIE 1931 chromaticity diagram of the Ho-SiAlON ceramics samples (**a**) upon 348 nm CW excitation (**b**) upon 348 nm pulsed excitation. Corresponding CIE coordinates are listed in Table 3. The emission modulation under CW and pulsed excitations result in different emission colors.

The emission spectra under CW (Figure 7a) and pulsed excitation (Figure 7b) are similar, but a slight change in emission intensity can be observed. The intensity modulation under CW and pulsed excitations can occur, and therefore, this results in slightly different emission colors, as can be seen from the CIE 1931 chromaticity diagram in Figure 8a,b. The CIE color coordinates presented in Table 3 under both the CW and pulsed excitations are in the green spectral region. Hence, Ho-SiAlON ceramics can be useful for green phosphors. A particular advantage of using Ho-SiAlON as green phosphor material is that there is no reduction of the emission intensity due to the concentration quenching, even at a very high level of Ho doping as shown above. On contrary, many other host materials, such as LaMgAl_11_O_19_ [40], Y_2_O_3_ [41], NaYF_4_ [13], K_2_SiF_6_ [12], etc., show a decrease in emission intensity due to concentration quenching at a high level of doping of the activators. An additional advantage of Ho-SiAlON ceramics as phosphors is that SiAlON ceramics have a very high laser damage threshold and high-temperature spectral stability [21,42] that make them suitable for high-power green emitting remote phosphors in extreme conditions [8,9].

**Figure 9:**
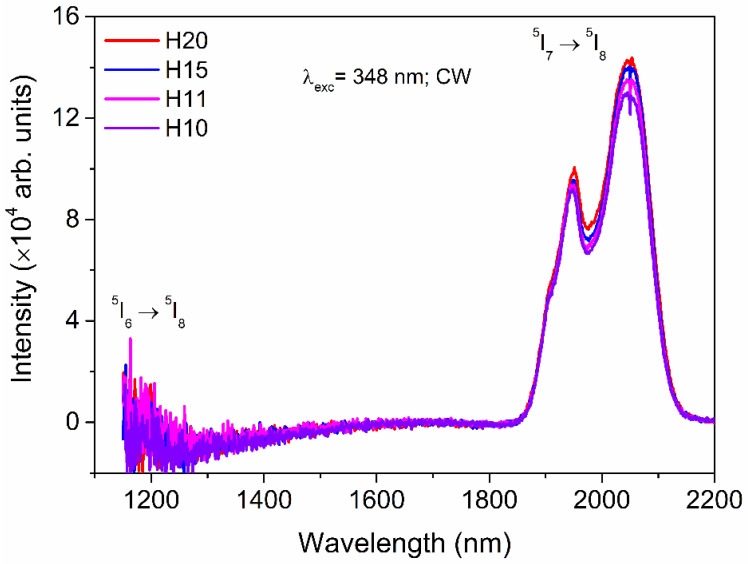
Downshifting spectra in the infrared range of Ho-SiAlON samples under 348 nm CW excitation.

Under 348 nm CW excitation, a broad mid-infrared emission band from 1850 to 2150 nm with FWHM of 186 nm is observed as shown in Figure 9, which can be assigned to ^5^I_7_ → ^5^I_8_ transition of Ho^3+^. The multiple peak profile of the emission band originates due to the rich Stark splitting of the ^5^I_7_ and ^5^I_8_ emitting states. A weak emission at 1196 nm corresponds to ^5^I_6_ → ^5^I_8_, whose Ho-concentration dependence is not evident, but there is a steady increase in the 2046 nm emission intensity with the increases in Ho concentration. The 2046 nm broadband emission of Ho^3+^ has a wide range of applications, such as in infrared lasers [43].

**Figure 10:**
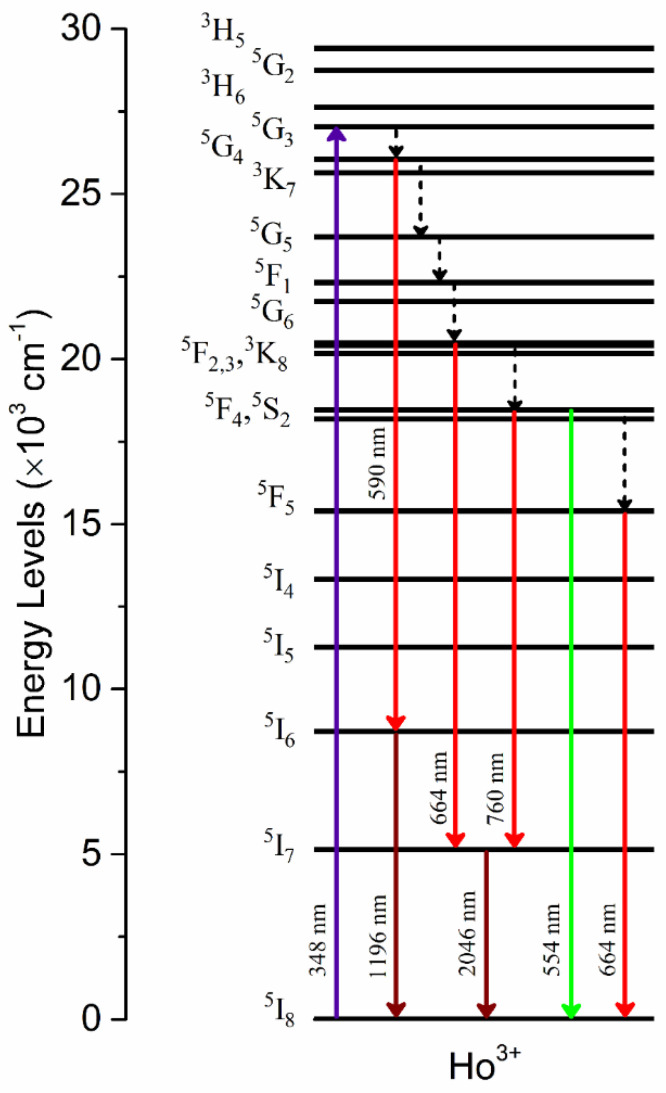
Energy level diagram of Ho^3+^ ion and involved downshifting photoluminescence mechanism under 348 nm excitation. Solid upward arrow represents absorption transition while downward arrows represent emission transitions. Dotted downward arrows represent non-radiative relaxation.

Downshifting emissions from Ho^3+^ under UV excitations have been reported for other crystal systems [44,45,46]. The observed downshifting emission properties of Ho-SiAlON under 348 nm excitation can be explained using the energy level diagram in Figure 10. Upon excitation at 348 nm, Ho^3+^ ions are first excited to the ^5^G_3_ manifold, and then via successive non-radiative relaxations, ^5^G_4_, ^5^F_2_,_3_, ^5^F_4_, and ^5^S_2_ manifolds are populated. A weak radiative transition at 590 nm appears from transition ^5^G_4_ → ^5^I_6_, which is followed by the ^5^I_6_ → ^5^I_8_ transition to produce another weak infrared emission at 1196 nm. However, strong red emissions at 664 and 760 nm are produced by the radiative transitions ^5^F_2_,_3_ → ^5^I_7_ and ^5^F_4_, ^5^S_2_ → ^5^I_7_, respectively. Both of these radiative transitions are followed by ^5^I_7_ → ^5^I_8_ to produce an intense infrared emission at 2046 nm. There are also other decay pathways of ^5^F_4_ and ^5^S_2_ manifolds to the ground state ^5^I_8_. Some of the Ho^3+^ ions in ^5^F_4_, ^5^S_2_ manifolds can directly undergo radiative decay to the ground state ^5^I_8_ to produce strong green emission at 554 nm, while other Ho^3+^ ions decay via the intermediated non-radiative relaxation ^5^F_4_, ^5^S_2_ → ^5^F_5_ to the ground state ^5^I_8_ via the decay to produce a strong red emission at 664 nm.

## 4. Conclusions

A series of Ho-SiAlON ceramics were prepared by the hot-press sintering method, and their photoluminescence properties were investigated under UV excitations. A fully dense α-SiAlON phase of all the sintered samples has been verified using XRD and TEM analysis. Moreover, the XPS analysis confirms that the Ho ions exist in a trivalent state. Ho-SiAlON ceramics show strong downshifting emissions under 348 nm excitation. Sharp and strong emission bands at 554 nm (green), 664 nm (red), 760 nm (near-infrared), and 2046 nm (infrared) broadband emission are observed as a result of the 4*f*-4*f* transition in Ho^3+^ ions. The intensity of the 554 nm emission band doubled on increasing the Ho concentration from 9.791 wt.% to 18.068 wt.% and no concentration quenching was observed. The CIE chromaticity color evaluation shows that Ho-SiAlON ceramics may be a potential candidate for green-emitting phosphors.

## Figures and Tables

**Table 1:** Starting m and n parameters, precursor compositions, and sample codes of the synthesized SiAlON samples.

Sample	m	n	Si_3_N_4_ (wt. %)	AlN (wt. %)	Al_2_O_3_ (wt. %)	Ho_2_O_3_ (wt. %)
H10	1.0	1.0	72.698	14.869	2.642	9.791
H11	1.1	1.1	70.792	15.619	2.888	10.701
H15	1.5	1.0	66.288	18.349	1.268	14.096
H20	2.0	1.0	60.372	21.560	0	18.068

**Table 2:** List of binding energy, full width at half maxima (FWHM), area, and proposed chemical-bond assignments. Integer identifiers are assigned with the lowest binding energy sub-peak as #1 to the sub-peaks within a given elemental peak. The unit of binding energy, FWHM, and the area are electron volts (eV).

Element	Peak	Binding Energy (eV)	FWHM (eV)	Area (eV)	Assignment
Si	Si2p #1	101.46	1.60	47,556.38	Si−N (Si_3_N_4_)
	Si2p #2	102.33	1.87	19,194.46	Si−O (Glassy grain boundary phase)
Al	Al2p #1	73.81	1.78	10,382.01	Al−N (AlN rich phase and grain boundaries)
	Al2p #2	74.39	1.98	2372.03	Al−O (Al(Si)–O(N) substitution in Si_3_N_4_)
O	O1s #1	531.93	2.31	114,724.90	O−Al (Al(Si)–O(N) substitution in Si_3_N_4_)
	O1s #2	532.39	1.28	14,043.52	O−Si (Glassy grain boundary phase)
N	N1s #1	396.20	1.73	10,604.92	N−Al (AlN rich phase and grain boundaries)
	N1s #2	397.21	1.64	117,564.10	N−Si (Si_3_N_4_)
	N1s #3	398.86	2.92	11,423.29	N−Ho
Ho	Ho4d #1	161.32	1.70	2843.22	4d_5/2_
	Ho4d #2	163.40	1.67	2309.50	4d_3/2_

**Table 3:** CIE coordinates of Ho-SiAlON ceramic samples under 348 CW and pulsed excitations.

Excitation Wavelength, Mode	CIE Diagram	Sample Name (Point on the CIE Diagram)	CIE Coordinates		
			X	Y	I (lum)
348 nm; CW	Figure 6a	H20 (1)	0.29663	0.55551	4.724 × 10^4^
		H15 (2)	0.30285	0.57289	3.338 × 10^4^
		H11 (3)	0.26377	0.47692	3.928 × 10^4^
		H10 (4)	0.31348	0.58476	2.533 × 10^4^
348 nm; Pulsed	Figure 6b	H20 (1)	0.34652	0.62740	1.124 × 10^3^
		H15 (2)	0.34571	0.63255	8.863 × 10^2^
		H11 (3)	0.33264	0.63021	1.094 × 10^3^
		H10 (4)	0.35148	0.62860	7.299 × 10^2^

## Data Availability

Not applicable.
